# 4-Bromo-2-[1-(4-eth­oxy­phen­yl)-1-methyl­eth­yl]-1-methyl­benzene

**DOI:** 10.1107/S1600536810049445

**Published:** 2010-12-04

**Authors:** Yan Li, Xue Kong, Guan-Hong Chen, Jian-Guo Chi, Jia-Ning Wang

**Affiliations:** a Biology Institute of Shandong Academy of Sciences, Jinan 250014, People’s Republic of China

## Abstract

In title compound, C_18_H_21_BrO, the dihedral angle between two rings is 85.72°. No classical hydrogen bonds are found and only van der Waals forces stabilize the crystal packing.

## Related literature

For details of the biological activity of SGLT2 inhibitors, a class of promising anti-hyperglycemic agents, see: Meng *et al.* (2008 ▶); Gao *et al.* (2010*a* ▶,*b* ▶). For bond-length data, see: Allen *et al.* (1987 ▶).
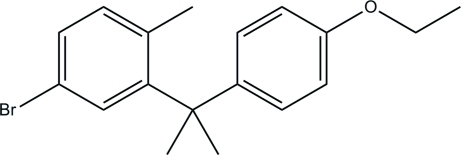


## Experimental

### 

#### Crystal data

C_18_H_21_BrO
*M* *_r_* = 333.26
Monoclinic, 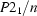

*a* = 11.164 (2) Å
*b* = 9.5142 (19) Å
*c* = 16.135 (3) Å
β = 110.21 (3)°
*V* = 1608.2 (6) Å^3^
*Z* = 4
Mo *K*α radiation
μ = 2.55 mm^−1^
*T* = 293 K
0.20 × 0.16 × 0.08 mm

#### Data collection

Rigaku Saturn diffractometer
Absorption correction: multi-scan (*CrystalClear*; Rigaku, 2005 ▶) *T*_min_ = 0.630, *T*_max_ = 0.822
13056 measured reflections
2839 independent reflections
2101 reflections with *I* > 2σ(*I*)
*R*_int_ = 0.054

#### Refinement

*R*[*F* ^2^ > 2σ(*F* ^2^)] = 0.051
*wR*(*F* ^2^) = 0.130
*S* = 1.05
2839 reflections
186 parameters
H-atom parameters constrained
Δρ_max_ = 0.52 e Å^−3^
Δρ_min_ = −0.39 e Å^−3^

### 

Data collection: *CrystalClear* (Rigaku, 2005 ▶); cell refinement: *CrystalClear*; data reduction: *CrystalClear*; program(s) used to solve structure: *SHELXTL* (Sheldrick, 2008 ▶); program(s) used to refine structure: *SHELXTL*; molecular graphics: *SHELXTL*; software used to prepare material for publication: *SHELXTL*.

## Supplementary Material

Crystal structure: contains datablocks I, global. DOI: 10.1107/S1600536810049445/hg2759sup1.cif

Structure factors: contains datablocks I. DOI: 10.1107/S1600536810049445/hg2759Isup2.hkl

Additional supplementary materials:  crystallographic information; 3D view; checkCIF report

## Figures and Tables

**Fig. 1.**
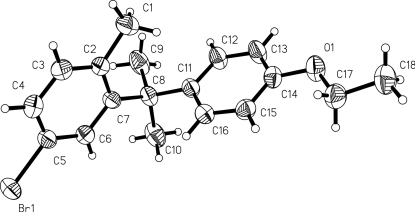
View of the title compound, with displacement ellipsoids drawn at the 40% probability level.

## supplementary crystallographic information

### Comment

SGLT2 inhibitors are a class of promising anti-hyperglycemic agents, and a variety of SGLT2 inhibitors are now in clinical trials (Meng *et al.*, 2008). The title compound was a crucial intermediate, the aglycon of the C-glucoside SGLT2 inhibitors, for the synthesis of novel C-glucoside SGLT2 inhibitors during the development of our own SGLT2 inhibitors (Gao *et al.*, 2010*a*,*b*).

In title compound, C_18_H_21_BrO, bond lengths are normal (Allen *et al.*, 1987). The dihedral angle between two phenyl ring (C2—C7 and C11—C16) is 85.72 °. No classic hydrogen bonds found, only Van der Waals forces stabilize the crystal packing.

### Experimental

A dried 100-ml round-bottomed flask was charged with 2.29 g (10 mmol) of 1-(5-bromo-2-methylphenyl)-1-methylethanol, 1.22 g (10 mmol) of phenetol and 15 ml of dried dichloromethane, and the clear solution thus obtained was stirred on an ice bath followed by addition of 1.33 g (10 mmol) of anhydrous aluminium chloride in a portionwise manner. After addition, the reaction mixture was stirred at room temperature for another one hour when TLC analysis indicated that all the starting materials were consumed completely. The reaction mixture was poured into 300 ml of ice-water and exacted with three 50-ml portions of dichloromethane, and the combined exacts were washed with saturated brine, dried over sodium sulfate and evaporated on a rotary evaporator to afford the crude product as a colorless oil, which was purified by column chromatography to yield the pure product as colorless crystals. Single crystals suitable for X-ray diffraction were obtained from slow evaporation of a solution of the pure title compound in dichloromethane/petroleum ether (1/4) at room temperature.

### Refinement

All H atoms were found on difference maps, with C—H = 0.93–0.97 and included in the final cycles of refinement using a riding model, with *U*_iso_(H) = 1.2*U*_eq_(C) and 1.5*U*_eq_(C) for the methyl H atoms.

### Crystal data

**Table:**

C_18_H_21_BrO	*F*(000) = 688
*M**_r_* = 333.26	*D*_x_ = 1.376 Mg m^−^^3^
Monoclinic, *P*2_1_/*n*	Mo *K*α radiation, λ = 0.71073 Å
Hall symbol: -P 2yn	Cell parameters from 3726 reflections
*a* = 11.164 (2) Å	θ = 2.1–27.1°
*b* = 9.5142 (19) Å	µ = 2.55 mm^−^^1^
*c* = 16.135 (3) Å	*T* = 293 K
β = 110.21 (3)°	Prism, colorless
*V* = 1608.2 (6) Å^3^	0.20 × 0.16 × 0.08 mm
*Z* = 4	

### Data collection

**Table:**

Rigaku Saturn diffractometer	2839 independent reflections
Radiation source: rotating anode	2101 reflections with *I* > 2σ(*I*)
confocal	*R*_int_ = 0.054
ω scans	θ_max_ = 25.0°, θ_min_ = 1.9°
Absorption correction: multi-scan (CystalClear; Rigaku, 2005)	*h* = −13→12
*T*_min_ = 0.630, *T*_max_ = 0.822	*k* = −11→11
13056 measured reflections	*l* = −17→19

### Refinement

**Table:**

Refinement on *F*^2^	Secondary atom site location: difference Fourier map
Least-squares matrix: full	Hydrogen site location: inferred from neighbouring sites
*R*[*F*^2^ > 2σ(*F*^2^)] = 0.051	H-atom parameters constrained
*wR*(*F*^2^) = 0.130	*w* = 1/[σ^2^(*F*_o_^2^) + (0.070*P*)^2^] where *P* = (*F*_o_^2^ + 2*F*_c_^2^)/3
*S* = 1.05	(Δ/σ)_max_ = 0.001
2839 reflections	Δρ_max_ = 0.52 e Å^−^^3^
186 parameters	Δρ_min_ = −0.39 e Å^−^^3^
0 restraints	Extinction correction: *SHELXTL* (Sheldrick, 2008), Fc^*^=kFc[1+0.001xFc^2^λ^3^/sin(2θ)]^-1/4^
Primary atom site location: structure-invariant direct methods	Extinction coefficient: 0.026 (2)

### Special details

**Table:**

Geometry. All e.s.d.'s (except the e.s.d. in the dihedral angle between two l.s. planes) are estimated using the full covariance matrix. The cell e.s.d.'s are taken into account individually in the estimation of e.s.d.'s in distances, angles and torsion angles; correlations between e.s.d.'s in cell parameters are only used when they are defined by crystal symmetry. An approximate (isotropic) treatment of cell e.s.d.'s is used for estimating e.s.d.'s involving l.s. planes.
Refinement. Refinement of *F*^2^ against ALL reflections. The weighted *R*-factor *wR* and goodness of fit *S* are based on *F*^2^, conventional *R*-factors *R* are based on *F*, with *F* set to zero for negative *F*^2^. The threshold expression of *F*^2^ > σ(*F*^2^) is used only for calculating *R*-factors(gt) *etc*. and is not relevant to the choice of reflections for refinement. *R*-factors based on *F*^2^ are statistically about twice as large as those based on *F*, and *R*- factors based on ALL data will be even larger.

### Fractional atomic coordinates and isotropic or equivalent isotropic displacement parameters (Å^2^)

**Table:**

	*x*	*y*	*z*	*U*_iso_*/*U*_eq_	
Br1	0.02586 (4)	0.72212 (4)	0.15240 (3)	0.0665 (3)	
O1	0.1869 (3)	−0.2600 (3)	0.39327 (19)	0.0644 (8)	
C1	−0.1877 (4)	0.1214 (4)	0.1522 (3)	0.0751 (13)	
H1A	−0.2276	0.0963	0.0912	0.113*	
H1B	−0.2496	0.1180	0.1812	0.113*	
H1C	−0.1198	0.0566	0.1802	0.113*	
C2	−0.1338 (4)	0.2691 (4)	0.1586 (3)	0.0545 (10)	
C3	−0.1463 (4)	0.3354 (5)	0.0805 (3)	0.0647 (11)	
H3	−0.1872	0.2878	0.0279	0.078*	
C4	−0.1010 (4)	0.4700 (4)	0.0762 (3)	0.0635 (11)	
H4	−0.1120	0.5129	0.0222	0.076*	
C5	−0.0388 (3)	0.5377 (4)	0.1551 (2)	0.0497 (9)	
C6	−0.0249 (3)	0.4749 (4)	0.2352 (2)	0.0491 (9)	
H6	0.0166	0.5236	0.2872	0.059*	
C7	−0.0716 (3)	0.3405 (4)	0.2397 (2)	0.0467 (8)	
C8	−0.0623 (4)	0.2769 (4)	0.3297 (3)	0.0501 (9)	
C9	−0.1980 (4)	0.2698 (4)	0.3348 (3)	0.0736 (13)	
H9A	−0.2450	0.1953	0.2975	0.110*	
H9B	−0.2410	0.3575	0.3153	0.110*	
H9C	−0.1921	0.2522	0.3946	0.110*	
C10	0.0203 (4)	0.3690 (4)	0.4079 (3)	0.0723 (12)	
H10A	0.0296	0.3226	0.4626	0.108*	
H10B	−0.0206	0.4583	0.4062	0.108*	
H10C	0.1029	0.3831	0.4034	0.108*	
C11	0.0025 (3)	0.1309 (4)	0.3436 (2)	0.0471 (9)	
C12	−0.0460 (4)	0.0150 (4)	0.3738 (2)	0.0575 (10)	
H12	−0.1226	0.0235	0.3843	0.069*	
C13	0.0171 (4)	−0.1130 (4)	0.3887 (3)	0.0628 (11)	
H13	−0.0187	−0.1895	0.4075	0.075*	
C14	0.1324 (4)	−0.1283 (4)	0.3759 (2)	0.0513 (9)	
C15	0.1832 (3)	−0.0141 (4)	0.3464 (2)	0.0525 (9)	
H15	0.2603	−0.0225	0.3367	0.063*	
C16	0.1182 (3)	0.1130 (4)	0.3314 (2)	0.0543 (10)	
H16	0.1539	0.1893	0.3123	0.065*	
C17	0.3065 (4)	−0.2787 (4)	0.3818 (3)	0.0673 (12)	
H17A	0.3699	−0.2168	0.4211	0.081*	
H17B	0.2990	−0.2568	0.3215	0.081*	
C18	0.3460 (4)	−0.4312 (5)	0.4026 (3)	0.0896 (15)	
H18A	0.3480	−0.4533	0.4611	0.134*	
H18B	0.4292	−0.4454	0.3991	0.134*	
H18C	0.2856	−0.4913	0.3608	0.134*	

### Atomic displacement parameters (Å^2^)

**Table:**

	*U*^11^	*U*^22^	*U*^33^	*U*^12^	*U*^13^	*U*^23^
Br1	0.0704 (4)	0.0471 (3)	0.0812 (4)	−0.00704 (19)	0.0251 (3)	0.01185 (19)
O1	0.068 (2)	0.0553 (17)	0.076 (2)	0.0164 (13)	0.0322 (16)	0.0155 (13)
C1	0.086 (3)	0.056 (3)	0.068 (3)	−0.020 (2)	0.009 (2)	−0.001 (2)
C2	0.054 (2)	0.042 (2)	0.055 (2)	−0.0056 (17)	0.004 (2)	0.0034 (17)
C3	0.068 (3)	0.055 (2)	0.056 (2)	−0.009 (2)	0.003 (2)	0.000 (2)
C4	0.063 (3)	0.065 (3)	0.054 (2)	0.001 (2)	0.008 (2)	0.012 (2)
C5	0.044 (2)	0.044 (2)	0.061 (2)	−0.0016 (16)	0.0181 (18)	0.0061 (17)
C6	0.050 (2)	0.046 (2)	0.050 (2)	−0.0002 (17)	0.0151 (17)	0.0002 (16)
C7	0.0421 (19)	0.039 (2)	0.058 (2)	−0.0014 (16)	0.0158 (17)	0.0028 (17)
C8	0.057 (2)	0.041 (2)	0.058 (2)	0.0041 (17)	0.0268 (19)	0.0062 (16)
C9	0.079 (3)	0.053 (3)	0.109 (4)	0.014 (2)	0.057 (3)	0.015 (2)
C10	0.114 (4)	0.054 (3)	0.053 (2)	−0.003 (2)	0.034 (2)	−0.0004 (19)
C11	0.053 (2)	0.042 (2)	0.047 (2)	−0.0014 (17)	0.0175 (17)	0.0036 (15)
C12	0.061 (2)	0.048 (2)	0.073 (3)	0.0085 (19)	0.037 (2)	0.0136 (19)
C13	0.072 (3)	0.046 (2)	0.080 (3)	0.006 (2)	0.037 (2)	0.018 (2)
C14	0.058 (2)	0.049 (2)	0.045 (2)	0.0076 (18)	0.0151 (18)	0.0053 (16)
C15	0.043 (2)	0.056 (2)	0.059 (2)	0.0039 (18)	0.0194 (18)	0.0040 (18)
C16	0.053 (2)	0.051 (2)	0.057 (2)	−0.0035 (18)	0.0159 (19)	0.0081 (17)
C17	0.066 (3)	0.059 (3)	0.073 (3)	0.009 (2)	0.020 (2)	−0.001 (2)
C18	0.082 (3)	0.064 (3)	0.120 (4)	0.024 (2)	0.032 (3)	0.007 (3)

### Geometric parameters (Å, °)

**Table:**

Br1—C5	1.904 (4)	C9—H9B	0.9600
O1—C14	1.379 (4)	C9—H9C	0.9600
O1—C17	1.421 (6)	C10—H10A	0.9600
C1—C2	1.518 (5)	C10—H10B	0.9600
C1—H1A	0.9600	C10—H10C	0.9600
C1—H1B	0.9600	C11—C16	1.384 (5)
C1—H1C	0.9600	C11—C12	1.389 (5)
C2—C3	1.373 (5)	C12—C13	1.386 (5)
C2—C7	1.423 (5)	C12—H12	0.9300
C3—C4	1.388 (6)	C13—C14	1.380 (5)
C3—H3	0.9300	C13—H13	0.9300
C4—C5	1.381 (5)	C14—C15	1.385 (5)
C4—H4	0.9300	C15—C16	1.388 (5)
C5—C6	1.382 (5)	C15—H15	0.9300
C6—C7	1.393 (5)	C16—H16	0.9300
C6—H6	0.9300	C17—C18	1.520 (5)
C7—C8	1.543 (5)	C17—H17A	0.9700
C8—C11	1.545 (5)	C17—H17B	0.9700
C8—C9	1.546 (6)	C18—H18A	0.9600
C8—C10	1.552 (6)	C18—H18B	0.9600
C9—H9A	0.9600	C18—H18C	0.9600
			
C14—O1—C17	117.1 (3)	C8—C10—H10A	109.5
C2—C1—H1A	109.5	C8—C10—H10B	109.5
C2—C1—H1B	109.5	H10A—C10—H10B	109.5
H1A—C1—H1B	109.5	C8—C10—H10C	109.5
C2—C1—H1C	109.5	H10A—C10—H10C	109.5
H1A—C1—H1C	109.5	H10B—C10—H10C	109.5
H1B—C1—H1C	109.5	C16—C11—C12	116.7 (3)
C3—C2—C7	119.2 (3)	C16—C11—C8	120.2 (3)
C3—C2—C1	116.8 (3)	C12—C11—C8	123.0 (3)
C7—C2—C1	124.1 (3)	C13—C12—C11	121.5 (3)
C2—C3—C4	123.2 (4)	C13—C12—H12	119.3
C2—C3—H3	118.4	C11—C12—H12	119.3
C4—C3—H3	118.4	C14—C13—C12	120.8 (4)
C5—C4—C3	117.4 (3)	C14—C13—H13	119.6
C5—C4—H4	121.3	C12—C13—H13	119.6
C3—C4—H4	121.3	O1—C14—C13	115.8 (3)
C4—C5—C6	121.3 (3)	O1—C14—C15	125.4 (3)
C4—C5—Br1	118.8 (3)	C13—C14—C15	118.9 (3)
C6—C5—Br1	119.9 (3)	C14—C15—C16	119.5 (3)
C5—C6—C7	121.5 (3)	C14—C15—H15	120.3
C5—C6—H6	119.3	C16—C15—H15	120.3
C7—C6—H6	119.3	C11—C16—C15	122.7 (3)
C6—C7—C2	117.6 (3)	C11—C16—H16	118.6
C6—C7—C8	120.3 (3)	C15—C16—H16	118.6
C2—C7—C8	122.0 (3)	O1—C17—C18	107.7 (4)
C7—C8—C11	111.5 (3)	O1—C17—H17A	110.2
C7—C8—C9	108.5 (3)	C18—C17—H17A	110.2
C11—C8—C9	111.8 (3)	O1—C17—H17B	110.2
C7—C8—C10	111.8 (3)	C18—C17—H17B	110.2
C11—C8—C10	105.8 (3)	H17A—C17—H17B	108.5
C9—C8—C10	107.4 (3)	C17—C18—H18A	109.5
C8—C9—H9A	109.5	C17—C18—H18B	109.5
C8—C9—H9B	109.5	H18A—C18—H18B	109.5
H9A—C9—H9B	109.5	C17—C18—H18C	109.5
C8—C9—H9C	109.5	H18A—C18—H18C	109.5
H9A—C9—H9C	109.5	H18B—C18—H18C	109.5
H9B—C9—H9C	109.5		
			
C7—C2—C3—C4	0.1 (6)	C7—C8—C11—C16	50.9 (5)
C1—C2—C3—C4	−179.6 (4)	C9—C8—C11—C16	172.5 (4)
C2—C3—C4—C5	1.0 (6)	C10—C8—C11—C16	−70.9 (4)
C3—C4—C5—C6	−1.4 (6)	C7—C8—C11—C12	−133.2 (4)
C3—C4—C5—Br1	179.7 (3)	C9—C8—C11—C12	−11.6 (5)
C4—C5—C6—C7	0.7 (5)	C10—C8—C11—C12	105.0 (4)
Br1—C5—C6—C7	179.6 (3)	C16—C11—C12—C13	−1.6 (6)
C5—C6—C7—C2	0.4 (5)	C8—C11—C12—C13	−177.6 (4)
C5—C6—C7—C8	−176.1 (3)	C11—C12—C13—C14	1.6 (6)
C3—C2—C7—C6	−0.8 (6)	C17—O1—C14—C13	−179.2 (4)
C1—C2—C7—C6	178.8 (4)	C17—O1—C14—C15	1.6 (5)
C3—C2—C7—C8	175.7 (4)	C12—C13—C14—O1	179.6 (4)
C1—C2—C7—C8	−4.7 (6)	C12—C13—C14—C15	−1.2 (6)
C6—C7—C8—C11	−127.0 (4)	O1—C14—C15—C16	179.9 (3)
C2—C7—C8—C11	56.7 (5)	C13—C14—C15—C16	0.8 (5)
C6—C7—C8—C9	109.5 (4)	C12—C11—C16—C15	1.2 (5)
C2—C7—C8—C9	−66.8 (4)	C8—C11—C16—C15	177.3 (3)
C6—C7—C8—C10	−8.8 (5)	C14—C15—C16—C11	−0.8 (6)
C2—C7—C8—C10	174.9 (4)	C14—O1—C17—C18	−178.8 (3)
